# A Second-Order Crank–Nicolson Leap-Frog Scheme for the Modified Phase Field Crystal Model with Long-Range Interaction

**DOI:** 10.3390/e24111512

**Published:** 2022-10-23

**Authors:** Chunya Wu, Xinlong Feng, Lingzhi Qian

**Affiliations:** 1 School of Mathematics and Statistics, Guangxi Normal University, Guilin 541006, China; 2 College of Mathematics and System Sciences, Xinjiang University, Urumqi 830046, China

**Keywords:** modified phase field crystal problem, Crank–Nicolson Leap-Frog, SAV method, second-order accuracy

## Abstract

In this paper, we construct a fully discrete and decoupled Crank–Nicolson Leap-Frog (CNLF) scheme for solving the modified phase field crystal model (MPFC) with long-range interaction. The idea of CNLF is to treat stiff terms implicity with Crank–Nicolson and to treat non-stiff terms explicitly with Leap-Frog. In addition, the scalar auxiliary variable (SAV) method is used to allow explicit treatment of the nonlinear potential, then, these technique combines with CNLF can lead to the highly efficient, fully decoupled and linear numerical scheme with constant coefficients at each time step. Furthermore, the Fourier spectral method is used for the spatial discretization. Finally, we show that the CNLF scheme is fully discrete, second-order decoupled and unconditionally stable. Ample numerical experiments in 2D and 3D are provided to demonstrate the accuracy, efficiency, and stability of the proposed method.

## 1. Introduction

It is well known that crystal phenomena (such as edge dislocation [1], deformation and plasticity in nanocrystalline materials [2], epitaxial growth, and zone refinement [3]) are usually described by phase-field crystal (PFC) models that preserve the original properties. The initial PFC model was presented by Elder and Grant [3,4], which mainly studied the growth dynamics of crystals at both spatial and time scales. In order to introduce a mechanism for simulating elastic interaction, the MPFC model was presented by Stefanovic et al. [5]. In addition, the MPFC model mainly describes the separate situation of time scales, which allows for the elastic relaxation of the crystal lattice on fast time scales, but other evolutionary processes are usually on slower time scales. The MPFC equation is given as follows:

(1) $$\begin{array}{cc} \; & \phi_{tt}+\beta \phi_{t}=M\Delta \mu ,\;\mathrm{in}\;\Omega \times \left(0,T\right],\\ \; & \mu ={\left(1+\Delta \right)}^{2}\phi +f\left(\phi \right), \end{array}$$

where Ω is a domain in $R^{d}\left(d=1,2,3\right)$, $f\left(\phi \right)=F^{\prime }\left(\phi \right)=\phi^{3}-\epsilon \phi$, 0<ϵ<1, ϕ is the density field, μ is the variation of *F* known as the chemical potential, M>0 is a mobility coefficient and β denotes a non-negative constant. The PFC model is obtained from the nonlocal free energy functional with respect to the gradient flows and the equilibrium state of system commonly is the minimum of the energy functional. Hence, the total free energy functional $E_{tol}\left(\phi \right)$ of the above governing Equation (1) is given as follows:

(2) $$\begin{array}{cc} \; & E_{tol}\left(\phi \right)=\int_{\Omega }\left(F\left(\phi \right)+\frac{1}{2}\phi {\left(1+\Delta \right)}^{2}\phi \right)dx,x\in \Omega . \end{array}$$

The energy functional $E_{tol}\left(\phi \right)$ decreases over time, i.e., the MPFC model satisfies the dissipation of energy law. In this study, we mainly focus on the MPFC model with long-range interaction, which is used to model the micro-phase separation process in diblock copolymers. It is well-known that the Ohta–Kawasaki (OK) model [6] mainly simulates the macro-phase separation process in diblock copolymer melts, which are chain molecules that makes up of two different and chemically incompatible segments connected by a covalent chemical bond. Therefore, on the basis of the Ohta–Kawasaki model, one can add a nonlocal operator that means the long-range interaction between the chain molecules. That is, we add a wave operator for the MPFC equation with long-range interaction. Nevertheless, it is challenging to design an accurate, easy-to-implement, efficient, and unconditionally stable scheme for this meaningful problem.

In this paper, we assume the periodic boundary condition and focus on time and space discretization. Therefore, we use the Fourier spectral method [7,8] for the spatial discretization for the system. In recent decades, many scholars focus on the numerical solution for the MPFC model and have conducted extensive related works and proposed various time-marching methods. For example, the semi-implicit method [9,10,11,12,13,14], the backward differentiation formula (BDF), and the Crank–Nicolson (CN) [15,16,17] method. In this paper, we mainly consider the CNLF method to deal with the MPFC model with long-range interaction. N. Hurl proved the stability of the unstable mode of the CNLF method, that is to say that CNLF does indeed control the unstable mode [18]. In order to eliminate the CFL time step retrictions of the CNLF scheme, N. Jiang proposed a new stabilized CNLF approach to the geophysical flow in [19,20], and the convergence and unconditional energy stability are proved in these works. Layton et al. [21] proposed a time-marching CNLF method for the uncoupling systems and demonstrated the conditional stability. The CNLF scheme was used to successfully solve geophysical flows, fast slow waves, and uncoupling groundwater–surface water flows in [22,23]. In addition, the CNLF scheme has some advantages, such as being a large, easy to decouple system, permitting the use of a large time step in actual numerical computations and parallel computation in decoupled sub-domain equations and avoiding some difficulties caused by nonlinear terms. Furthermore, we add a wave operator to the MPFC equation with long-range interaction, which is more beneficial to solving the CNLF scheme.

In recent years, it has become extremely important to develop efficient and easy-to-implement algorithms for gradient flow models. There are some popular strategies, for example, the convex splitting (CS) [24,25] methods, the invariant energy quadratization (IEQ) [26,27] and scalar auxiliary variable(SAV) [28,29,30] methods, etc. One can design first- and second-order unconditionally energy stable schemes by using the CS method; however, the second-order scheme of the CS method is nonlinear and implicit, which can potentially cause difficulties in numerical computation and some normally iterative steps. The IEQ method requires solving some coupled linear equations with complicated variable coefficients at each time step, which leads to high computational costs. Compared with the above two methods, the SAV method is more efficient and easy to implement, as it just needs to solve a purely linear system with constant coefficients at each time step. In this paper, we derive an equivalent PDE system by replacing the original variable with some new auxiliary variables. After developing a second-order CNLF scheme for the reformulated PDE system, we only need three steps to deal with two fully decoupled, sixth-order, purely linear equations for the MPFC equation with long-range interaction.

The main goal of this work is to construct a CNLF approach for solving the MPFC model with long-range interaction and prove the properties of the CNLF scheme. Our main contributions are:

- We construct a second-order decoupled CNLF scheme by combining it with the SAV method in time and the Fourier spectral method in space and carry out the unique solvability, mass conservation, and stability for the MPFC equation with long-range interaction.
- The presented scheme is very easy to implement and highly efficient. We only need to solve two six-order, fully decoupled and linear equations with some constant coefficients at each time step.
- We compare the second-order accuracy and errors of the presented scheme with the CN and BDF2 schemes numerically. In addition, we display the dynamic evolution of the energy of phase variable in experiments with long-range interaction.

The rest of this paper can be split into four sections. In Section 2, we introduce the governing systems and state the corresponding energy dissipation law. In Section 3, we design a second-order fully discrete and decoupled CNLF scheme by applying the SAV approach in time and the Fourier spectral method in space for the MPFC model with long-range interaction. The mass conservation and unconditional stability of the proposed scheme are proven rigorously, and we demonstrate the uniform-in-time $H^{2}$ bound for the numerical solution. In Section 4, the effectiveness and unconditional stability of the proposed method are depicted through various numerical experiments. Finally, conclusions can be described in Section 5.

## 2. Governing Systems

In this section, we focus on the following MPFC model with long-range interaction [17]:

(3) $$\begin{array}{cc} \; & \tilde{\beta }\phi_{tt}+\beta \phi_{t}=M\left(\Delta \mu -\sigma (\phi -\bar{\phi })\right),\mathrm{in}\;\Omega \times \left(0,T\right].\\ \; & \mu ={\left(1+\Delta \right)}^{2}\phi +f\left(\phi \right), \end{array}$$

which satisfies the initial condition ${\phi |}_{t=0}=\phi^{0}\left(x\right)$ and $\phi_{t}{|}_{t=0}=0$. In addition, $\Omega \subset R^{d}\left(d=1,2,3\right)$, $f\left(\phi \right)=\phi^{3}-\epsilon \phi$. Let us define $\bar{\phi }$ as the mean value of ϕ over Ω, i.e., $\bar{\phi }=\frac{1}{\left|\Omega \right|}\int_{\Omega }\phi dx$. $\tilde{\beta }$ and β are two positive parameters, and σ denotes the strength of the long-range interaction. If σ=0 and $\tilde{\beta }=0$, the above model is the classical PFC model. If σ≠0, $\sigma (\phi -\bar{\phi })$ is a nonlocal potential term. The nonlocal term is derived from the following second term of the free energy functional E(ϕ):

(4) $$\begin{array}{c} E\left(\phi \right)=\underset{I}{\underset{︸}{\int_{\Omega }\left(\frac{1}{2}\phi {\left(1+\Delta \right)}^{2}\phi +F\left(\phi \right)\right)dx}}+\underset{II}{\underset{︸}{\frac{\sigma }{2}\int_{\Omega }\int_{\Omega }G\left(x-y\right)\left(\phi \left(x\right)-\bar{\phi }\right)\left(\phi \left(y\right)-\bar{\phi }\right)dxdy}}, \end{array}$$

where the first term is the so-called Swift–Hohenberg-type [31] functional and the second term is the nonlocal potential with long-range interaction.

We first give some notations here. Let (·,·) and ∥·∥ denote the standard $L^{2}$ inner product and the corresponding induced $L^{2}$ norm. For each s≥0, we use ${\left(\cdot ,\cdot \right)}_{s}$ and ${∥\cdot ∥}_{s}$ to denote the inner product and norm in $H^{s}\left(\Omega \right)$. Note that $H^{0}\left(\Omega \right)=L^{2}\left(\Omega \right)$. We define Hilbert spaces $L_{0}^{2}\left(\Omega \right)=\left\{u\in L^{2}\left(\Omega \right)|\left(u,1\right)=0\right\}$, $L_{per}^{2}\left(\Omega \right)=\left\{u\in L^{2}\left(\Omega \right)|u\;\mathrm{is}\;\mathrm{periodic}\;\mathrm{on}\;\partial \Omega \right\}$, $H_{per}^{s}=\left\{u\in H^{s}\left(\Omega \right)|u\;\mathrm{is}\;\mathrm{periodic}\;\mathrm{on}\;\partial \Omega \right\}$, and $H_{per}^{-s}\left(\Omega \right)$ is the dual space of $H_{per}^{s}\left(\Omega \right)$. Then, let us introduce the inverse Laplace operator ${\left(-\Delta \right)}^{-1}$ and the $H_{per}^{-1}$ inner product. Suppose $f\in L_{0}^{2}\left(\Omega \right)$ and let $u\in H_{per}^{2}\left(\Omega \right)\cap L_{0}^{2}\left(\Omega \right)$ be the solution of the periodic boundary value problem:

(5) −Δu=finΩ.

Then, we define $u={\left(-\Delta \right)}^{-1}f$. Given $f,g\in L_{0}^{2}\left(\Omega \right)$, the $H_{per}^{-1}$ inner product can be defined by:

(6) $${\left(f,g\right)}_{-1}=\left(\nabla u_{1},\nabla u_{2}\right),$$

where $u_{1},u_{2}\in H_{per}^{2}\left(\Omega \right)\cap L_{0}^{2}\left(\Omega \right)$ are the solutions of the periodic boundary value problems $-\Delta u_{1}=f$ and $-\Delta u_{2}=g$, respectively. The $H^{-1}$ norm is defined by ${∥f∥}_{-1}=\sqrt{{\left(f,f\right)}_{-1}}$. We can easily obtain:

(7) $$\begin{array}{c} {\left(f,g\right)}_{-1}=\left({\left(-\Delta \right)}^{-1}f,g\right)=\left({\left(-\Delta \right)}^{-\frac{1}{2}}f,{\left(-\Delta \right)}^{-\frac{1}{2}}g\right)=\left({\left(-\Delta \right)}^{-1}g,f\right)={\left(g,f\right)}_{-1}. \end{array}$$

In Formula (4), *G* denotes the Green’s function of −Δ with periodic boundary conditions, which has the property of ΔG(x−y)=−δ(x−y) [32]. Thus, we note that the second term of E(ϕ) can be rewritten as follows:

(8) $$\begin{array}{cc} \; & \frac{\sigma }{2}\int_{\Omega }\int_{\Omega }G\left(x-y\right)\left(\phi \left(x\right)-\bar{\phi }\right)\left(\phi \left(y\right)-\bar{\phi }\right)dxdy\\ \; & =\frac{\sigma }{2}\int_{\Omega }{\left(-\Delta \right)}^{-1}\left(\phi \left(x\right)-\bar{\phi }\right)\left(\int_{\Omega }-\Delta G\left(x-y\right)\left(\phi \left(y\right)-\bar{\phi }\right)dy\right)dx\\ \; & =\frac{\sigma }{2}\int_{\Omega }{|\left(-\Delta \right)}^{-\frac{1}{2}}\left(\phi \left(x\right)-\bar{\phi }\right){|}^{2}dx=\frac{\sigma }{2}{∥\phi -\bar{\phi }∥}_{-1}^{2}, \end{array}$$

and then (4) can be rewritten as:

(9) $$\begin{array}{c} E\left(\phi \right)=\int_{\Omega }\left(\frac{1}{2}\phi {\left(1+\Delta \right)}^{2}\phi +F\left(\phi \right)\right)dx+\frac{\sigma }{2}{∥\phi -\bar{\phi }∥}_{-1}^{2}. \end{array}$$

The MPFC model with long-range interaction (3) satisfies the following two properties.

**Lemma** **1.** *Mass conservation: assuming that the initial condition satisfies* $\phi_{t}\left(x,0\right)=0$ *, we have:*

(10) $$\begin{array}{c} \int_{\Omega }\phi_{t}\left(x,t\right)dx=\int_{\Omega }\phi_{tt}\left(x,t\right)dx=0. \end{array}$$

**Proof.**  By integrating the first equation of (3) over Ω with the periodic boundary condition for μ, we derive:

(11) $$\begin{array}{c} \tilde{\beta }\frac{d}{dt}\int_{\Omega }\phi_{t}\left(x,t\right)dx+\beta \int_{\Omega }\phi_{t}\left(x,t\right)dx=M\int_{\partial \Omega }\nabla \mu \cdot nds=0. \end{array}$$

Then, the solution of the Equation (11) is:

(12) $$\begin{array}{c} \int_{\Omega }\phi_{t}\left(x,t\right)dx=e^{-\beta t/\tilde{\beta }}\int_{\Omega }\phi_{t}\left(x,0\right)dx. \end{array}$$

Therefore, as long as the initial condition satisfies $\phi_{t}\left(x,0\right)=0$, then $\int_{\Omega }\phi_{t}\left(x,t\right)dx=0$, and we also have:

(13) $$\begin{array}{c} \int_{\Omega }\phi_{tt}\left(x,t\right)dx=0. \end{array}$$

Therefore, we complete the proof. □

**Lemma** **2** ([17])**.** *We introduce a new variable $\psi =\phi_{t}$. The governing model follows an energy dissipation law:*

(14) $$\begin{array}{cc} \; & \frac{d}{dt}\varepsilon \left(\phi ,\psi \right)=-\frac{\beta }{M}{∥\psi ∥}_{-1}^{2}\leq 0, \end{array}$$

*where the pseudo energy ε(ϕ,ψ) is defined as follows:*

(15) $$\begin{array}{cc} \; & \varepsilon \left(\phi ,\psi \right):=E\left(\phi \right)+\frac{\tilde{\beta }}{2M}{∥\psi ∥}_{-1}^{2}. \end{array}$$

**Proof.**  Obviously, $\psi ,\psi_{t}\in L_{0}^{2}\left(\Omega \right)$. Utilizing the operator $\Delta^{-1}$, we can rewrite the system (3) as follows:

(16) $$\begin{array}{c} \tilde{\beta }\Delta^{-1}\psi_{t}+\beta \Delta^{-1}\psi =M\left({\left(1+\Delta \right)}^{2}\phi +f\left(\phi \right)-\sigma \Delta^{-1}\left(\phi -\bar{\phi }\right)\right), \end{array}$$

and by taking the $L^{2}$ inner product of (16) with $\frac{1}{M}\phi_{t}$, we obtain:

(17) $$\begin{array}{cc} \; & E\left(\phi \right)+\frac{\tilde{\beta }}{2M}{∥\psi ∥}_{-1}^{2}=-\frac{\beta }{M}{∥\psi ∥}_{-1}^{2}\leq 0, \end{array}$$

This implies that the pseudo energy ε(ϕ,ψ) of system (3) is non-increasing in time. □

## 3. Numerical Scheme

In this section, we firstly convert system (3) to an equivalent form by employing the SAV approach, which aims to transform the nonlinear term of total energy into a simple quadratic form in terms of some new auxiliary variables, and we can also demonstrate the energy stability of the new equivalent system. To this end, two auxiliary variables are introduced as follows:

(18) $$\begin{array}{c} Q=\Delta^{-1}\psi ,\;U=\sqrt{E_{1}\left(\phi \right)+B_{0}}, \end{array}$$

where $E_{1}\left(\phi \right):=\int_{\Omega }F\left(\phi \right)dx$, and $B_{0}$ denotes a non-negative constant to ensure $E_{1}\left(\phi \right)+B_{0}>0$. Therefore, the free energy functional is rewritten as:

(19) $$\begin{array}{c} \tilde{E}\left(\phi ,U\right)=\int_{\Omega }\left(\frac{1}{2}\phi {\left(1+\Delta \right)}^{2}\phi +\frac{\sigma }{2}{\left|{\left(-\Delta \right)}^{-\frac{1}{2}}\left(\phi -\bar{\phi }\right)\right|}^{2}\right)dx+U^{2}-B_{0}, \end{array}$$

and the pseudo energy is obtained as:

(20) $$\begin{array}{c} \hat{\varepsilon }\left(\phi ,U,Q\right)=\tilde{E}\left(\phi ,U\right)+\frac{\tilde{\beta }}{2M}{∥\nabla Q∥}^{2}. \end{array}$$

Now, a new but equivalent PDE system is derived as follows:

(21) $$\begin{array}{cc} \; & \tilde{\beta }\psi_{t}+\beta \psi =M\left(\Delta \mu -\sigma (\phi -\bar{\phi })\right), \end{array}$$

(22) $$\begin{array}{cc} \; & \mu ={\left(1+\Delta \right)}^{2}\phi +H\left(\phi \right)U, \end{array}$$

(23) $$\begin{array}{cc} \; & \psi =\phi_{t}, \end{array}$$

(24) $$\begin{array}{cc} \; & U_{t}=\frac{1}{2}\int_{\Omega }H\left(\phi \right)\phi_{t}dx, \end{array}$$

where $H\left(\phi \right)=f\left(\phi \right)/\sqrt{E_{1}\left(\phi \right)+B_{0}}$. The variables are equipped with the compatible initial boundary conditions:

(25) $$\begin{array}{c} {\phi |}_{t=0}=\phi^{0},\psi {{|}_{t=0}=0,U|}_{t=0}=\sqrt{E_{1}\left(\phi^{0}\right)+B_{0}}. \end{array}$$

It is worth noting that *U* depends only on time; thus, *U* does not require any boundary conditions, and other variables adopt periodic boundary conditions. Furthermore, the system (21)–(24) still keeps the energy dissipation law:

(26) $$\begin{array}{c} \frac{d}{dt}\hat{\varepsilon }\left(\phi ,U,Q\right)=-\frac{\beta }{M}{∥\nabla Q∥}^{2}\leq 0. \end{array}$$

**Remark** **1.** *It should be noted that system (21)–(24) is equivalent to the original system (3) with the same initial conditions. The energy (20) and its dissipation law (26) for the system (21)–(24) are exactly the same as the original energy (3) and its dissipation law (14), respectively. Then, in the following subsection, we will construct an efficient and fully discrete numerical scheme for the transformed system (21)–(24).*

### 3.1. The Semi-Discrete CNLF Scheme

In this subsection, we design a second-order, time-marching scheme to deal with the MPFC problem with long-range interaction by employing the SAV method. In the following, we use δt>0 to denote the time step and set $t^{n}=n\delta t$ for 0≤n≤N with the final time T=Nδt. Assuming that $\left(\phi^{n-1},\psi^{n-1},U^{n-1}\right)$ and $\left(\phi^{n},\psi^{n},U^{n}\right)$ are already calculated with n≥1, we update $\left(\phi^{n+1},\psi^{n+1},U^{n+1}\right)$:

(27) $$\begin{array}{cc} \; & \tilde{\beta }\frac{\psi^{n+1}-\psi^{n-1}}{2\delta t}+\beta \frac{\psi^{n+1}+\psi^{n-1}}{2}=M\left(\Delta \mu^{n}-\sigma \left(\frac{\phi^{n+1}+\phi^{n-1}}{2}-\frac{{\bar{\phi }}^{n+1}+{\bar{\phi }}^{n-1}}{2}\right)\right), \end{array}$$

(28) $$\begin{array}{cc} \; & \mu^{n}={\left(1+\Delta \right)}^{2}\frac{\phi^{n+1}+\phi^{n-1}}{2}+H\left(\phi^{n}\right)\frac{U^{n+1}+U^{n-1}}{2}, \end{array}$$

(29) $$\begin{array}{cc} \; & \frac{\psi^{n+1}+\psi^{n-1}}{2}=\frac{\phi^{n+1}-\phi^{n-1}}{2\delta t}, \end{array}$$

(30) $$\begin{array}{cc} \; & \frac{U^{n+1}-U^{n-1}}{2\delta t}=\frac{1}{2}\int_{\Omega }H\left(\phi^{n}\right)\frac{\phi^{n+1}-\phi^{n-1}}{2\delta t}dx, \end{array}$$

where ${\bar{\phi }}^{n+1}=\frac{1}{\left|\Omega \right|}\int_{\Omega }\phi^{n+1}dx$ and the initial conditions ${\phi |}_{t=0}=\phi^{0}{,\psi |}_{t=0}=0$ and ${U|}_{t=0}=\sqrt{E\left(\phi^{0}\right)+B_{0}}$.

**Theorem** **1.** *The linear system resulting from the scheme (27)–(30) is uniquely solvable.*

**Proof.**  We firstly rewrite (29) and (30) as:

(31) $$\begin{array}{l} U^{n+1}=\frac{1}{2}\left(H\left(\phi^{n}\right),\phi^{n+1}\right)+U^{n-1}-\frac{1}{2}\left(H\left(\phi^{n}\right),\phi^{n-1}\right), \end{array}$$

(32) $$\begin{array}{l} \psi^{n+1}=\frac{\phi^{n+1}}{\delta t}-\frac{\phi^{n-1}}{\delta t}-\psi^{n-1}. \end{array}$$

Then, (27) and (28) are rewritten as follows:

(33) $$\begin{array}{l} \left(\frac{\tilde{\beta }}{\delta t}+\beta \right)\phi^{n+1}=\tilde{\beta }\left(\frac{\phi^{n-1}}{\delta t}+2\psi^{n-1}\right)+\beta \phi^{n-1}+2\delta tM\left(\Delta \mu^{n}-\sigma \left(\frac{\phi^{n+1}+\phi^{n-1}}{2}-\frac{{\bar{\phi }}^{n+1}+{\bar{\phi }}^{n-1}}{2}\right)\right), \end{array}$$

(34) $$\begin{array}{l} \mu^{n}={\left(1+\Delta \right)}^{2}\frac{\phi^{n+1}+\phi^{n-1}}{2}+\frac{1}{2}H\left(\phi^{n}\right)(\frac{1}{2}\left(H\left(\phi^{n}\right),\phi^{n+1}\right)+2U^{n-1}-\frac{1}{2}\left(H\left(\phi^{n}\right),\phi^{n-1}\right)). \end{array}$$

Through taking the integration of (33) over Ω, we can easily derive:

(35) $$\begin{array}{c} {\bar{\phi }}^{n+1}+{\bar{\phi }}^{n-1}=\frac{\delta t}{\tilde{\beta }+\delta t\beta }\frac{1}{\left|\Omega \right|}\int_{\Omega }\left(\tilde{\beta }\left(\frac{\phi^{n-1}}{\delta t}+2\psi^{n-1}\right)+\left(\frac{\tilde{\beta }}{\delta t}+2\beta \right)\phi^{n-1}\right)dx. \end{array}$$

Thus, the presented scheme can be rewritten equivalently as follows:

(36) $$\begin{array}{cc} \; & ℘\left(\phi^{n+1}\right)-\frac{1}{2}\delta tM\Delta H\left(\phi^{n}\right)(H\left(\phi^{n}\right),\phi^{n+1})=q^{n-1}, \end{array}$$

where

(37) $$\begin{array}{cc} & ℘\left(\phi^{n+1}\right)=\left(\frac{\tilde{\beta }}{\delta t}+\beta \right)\phi^{n+1}-\delta tM\Delta {\left(1+\Delta \right)}^{2}\phi^{n+1}+\delta tM\sigma \phi^{n+1}, \end{array}$$

(38) $$\begin{array}{cc} & q^{n-1}=\tilde{\beta }\left(\frac{\phi^{n-1}}{\delta t}+2\psi^{n-1}\right)+\delta tM\Delta {\left(1+\Delta \right)}^{2}\phi^{n-1}+\delta tM\Delta H\left(\phi^{n}\right)\left(2U^{n-1}-\frac{1}{2}\left(H\left(\phi^{n}\right),\phi^{n-1}\right)\right)\\ & +\beta \phi^{n-1}-\delta tM\sigma \phi^{n-1}+\delta tM\sigma \frac{\delta t}{\tilde{\beta }+\delta t\beta }\frac{1}{\left|\Omega \right|}\int_{\Omega }\left(\tilde{\beta }\left(\frac{\phi^{n-1}}{\delta t}+2\psi^{n-1}\right)+\left(\frac{\tilde{\beta }}{\delta t}+2\beta \right)\phi^{n-1}\right)dx. \end{array}$$

Next, we need to compute $\left(H\left(\phi^{n}\right),\phi^{n+1}\right)$ before considering the existence and uniqueness of (36). Multiplying (36) with ${\left(\frac{\tilde{\beta }}{\delta t}+\beta -\delta tM\Delta {\left(1+\Delta \right)}^{2}+\delta tM\sigma \right)}^{-1}:={℘}^{-1}$ and taking the inner product with $H(\phi^{n})$, we obtain:

(39) $$\begin{array}{cc} \; & (H\left(\phi^{n}\right),\phi^{n+1})+\frac{\delta tM}{2}\gamma^{n}(H\left(\phi^{n}\right),\phi^{n+1})=(H\left(\phi^{n}\right),{℘}^{-1}\left(q^{n-1}\right)), \end{array}$$

where $\gamma^{n}=-\left(H\left(\phi^{n}\right),{℘}^{-1}\left(\Delta H\left(\phi^{n}\right)\right)\right)$. Then, we prove that *℘* is a symmetric positive definite operator. Since for any $\phi_{1}$ and $\phi_{2}$ with the periodic boundary condition we have:

(40) $$\begin{array}{cc} \left(℘\left(\phi_{1}\right),\phi_{2}\right) & =\left(\left(\frac{\tilde{\beta }}{\delta t}+\beta -\delta tM\Delta {\left(1+\Delta \right)}^{2}+\delta tM\sigma \right)\phi_{1},\phi_{2}\right)\\ \; & =\left(\phi_{1},\left(\frac{\tilde{\beta }}{\delta t}+\beta -\delta tM\Delta {\left(1+\Delta \right)}^{2}+\delta tM\sigma \right)\phi_{2}\right)\\ \; & =(\phi_{1},℘\left(\phi_{2}\right)), \end{array}$$

this means that the operator *℘* is symmetric.

In addition, it is easy to derive that for any ϕ with the periodic boundary condition, we have

(41) $$\begin{array}{cc} \left(℘(\phi ),\phi \right) & =\left((\frac{\tilde{\beta }}{\delta t}+\beta -\delta tM\Delta {\left(1+\Delta \right)}^{2}+\delta tM\sigma )\phi ,\phi \right)\\ \; & =\left(\frac{\tilde{\beta }}{\delta t}+\beta \right){∥\phi ∥}^{2}+\delta tM∥\nabla \left(1+\Delta \right){\phi ∥}^{2}+\delta tM\sigma {∥\phi ∥}^{2}\geq 0. \end{array}$$

Therefore, we have the unique solution:

(42) $$\begin{array}{cc} \; & (H\left(\phi^{n}\right),\phi^{n+1})=\frac{\left(H\left(\phi^{n}\right),{℘}^{-1}\left(q^{n-1}\right)\right)}{1+\frac{\delta tM}{2}\gamma^{n}}, \end{array}$$

where $\gamma^{n}\geq 0$, since all the eigenvalues of the symmetric operator ${℘}^{-1}\left(\Delta H\left(\phi^{n}\right)\right)$ are non-positive. Then, substituting (42) into (36) leads to:

(43) $$\begin{array}{cc} \; & ℘\left(\phi^{n+1}\right)=q^{n-1}+\frac{\delta tM\Delta H\left(\phi^{n}\right)\left({℘}^{-1}\left(q^{n-1}\right),H\left(\phi^{n}\right)\right)}{2-\delta tM({℘}^{-1}\left(\Delta H\left(\phi^{n}\right)\right),H\left(\phi^{n}\right))}, \end{array}$$

which implies the existence and uniqueness of the scheme (27)–(30) due to the fact that *℘* is a symmetric positive definite operator.

Furthermore, it is very easy to solve the scheme (27)–(30) in the following procedure:

- Compute $\gamma^{n}=-\left(H\left(\phi^{n}\right),{℘}^{-1}\left(\Delta H\left(\phi^{n}\right)\right)\right)$ by solving a six-order decoupled linear equation $\nu_{1}={℘}^{-1}\left(\Delta H(\phi^{n})\right)$;
- Compute $\left(H\left(\phi^{n}\right),\phi^{n+1}\right)$ from (42) and $U^{n+1}$ from (30) by solving another six-order decoupled linear equation $\nu_{2}={℘}^{-1}\left(q^{n-1}\right)$;
- Finally, based on the above two steps, we can obtain $\phi^{n+1}$ from (37) as:
  (44) $$\begin{array}{c} \phi^{n+1}=\frac{\delta tM}{2}\left(H\left(\phi^{n}\right),\phi^{n+1}\right)\nu_{1}+\nu_{2}. \end{array}$$

 □

Therefore, we just need to compute ${℘}^{-1}\left(\Delta H\left(\phi^{n}\right)\right)$ and ${℘}^{-1}\left(q^{n-1}\right)$; in other words, the total price of computing the CNLF scheme (27)–(30) at each time step is merely need to solve two completely decoupled, six-order, purely linear equations with the periodic boundary conditions.

**Remark** **2.** *The above solution processes are similar to reference [17]. Since the linear operator ℘ is a constant coefficient matrix, we show that ℘ is symmetric and positive definite, and it also follows that the operator ${℘}^{-1}\left(\Delta \left(\cdot \right)\right)$ is non-positive. Thus, the existence and uniqueness of formula (36) are obtained. In addition, a detailed explanation of the operator ${℘}^{-1}$ is provided in reference [17], and we leave it to the interested readers.*

**Remark** **3.** *In this paper, we rigorously demonstrate the unique solvability of the scheme (27)–(30) at the semi-discrete level. For the fully discrete scheme, we could be proven with similar analysis in Theorem 1. We leave the detailed proof of the unique solvability to the interested reader.*

### 3.2. The Fully Discrete CNLF Scheme with Fourier Spectral Method in Space

We describe the Fourier spectral framework [7]. We denote the domain $\Omega =\left(0,L_{x}\right)\times \left(0,L_{y}\right)$ uniformly with $h_{x}=L_{x}/N_{x},h_{y}=L_{y}/N_{y}$, where $N_{x}$ and $N_{y}$ are positive integers. The Fourier approximation space is:

(45) $$S_{h}=span\left\{e^{i\zeta_{k}x}e^{i\eta_{l}y}:-\frac{N_{x}}{2}\leq k\leq \frac{N_{x}}{2}-1,-\frac{N_{y}}{2}\leq l\leq \frac{N_{y}}{2}-1\right\},$$

where $i=\sqrt{-1},\zeta_{k}=2\pi k/L_{x},\eta_{l}=2\pi l/L_{y}$. Then, any function $u\left(x,y\right)\in L^{2}\left(\Omega \right)$ can be approximated by:

(46) $$u\left(x,y\right)\approx u_{h}\left(x,y\right)=\sum_{k=-\frac{N_{x}}{2}}^{\frac{N_{x}}{2}-1}\sum_{k=-\frac{N_{y}}{2}}^{\frac{N_{y}}{2}-1}{\hat{u}}_{k,l}e^{i\zeta_{k}x}e^{i\eta_{l}y},$$

where the Fourier coefficients are denoted:

(47) $${\hat{u}}_{k,l}=\langle u,e^{i\zeta_{k}x}e^{i\eta_{l}y}\rangle =\frac{1}{\Omega }\int_{\left|\Omega \right|}ue^{-i(\zeta_{k}x+\eta_{l}y)}dx.$$

In what follows, we take $N=N_{x}=N_{y}$ for simplicity. The full discrete scheme with the Fourier spectral method in space can be constructed as follows: Assuming $\phi_{h}^{n},\psi_{h}^{n},U^{n}$ and $\phi_{h}^{n-1},\psi_{h}^{n-1},U^{n-1}$ are known, and for ∀Θ,Λ and $\Upsilon \in S_{h}$, we update $\phi_{h}^{n+1},\psi_{h}^{n+1},U^{n+1}$ by solving

(48) $$\begin{array}{cc} \; & \tilde{\beta }\left(\frac{\psi_{h}^{n+1}-\psi_{h}^{n-1}}{2\delta t},\Theta \right)+\beta \left(\frac{\psi_{h}^{n+1}+\psi_{h}^{n-1}}{2},\Theta \right)=M\left(\Delta \mu_{h}^{n},\Theta \right)-M\sigma \left((\frac{\phi_{h}^{n+1}+\phi_{h}^{n-1}}{2}-\frac{{\bar{\phi }}_{h}^{n+1}+{\bar{\phi }}_{h}^{n-1}}{2}),\Theta \right), \end{array}$$

(49) $$\begin{array}{cc} \; & \left(\mu_{h}^{n},\Lambda \right)=\left(\left(1+\Delta \right)\frac{\phi_{h}^{n+1}+\phi_{h}^{n-1}}{2},\left(1+\Delta \right)\Lambda \right)+\left(H\left(\phi_{h}^{n}\right)\frac{U^{n+1}+U^{n-1}}{2},\Lambda \right), \end{array}$$

(50) $$\begin{array}{cc} \; & \left(\frac{\psi_{h}^{n+1}+\psi_{h}^{n-1}}{2},\Upsilon \right)=\left(\frac{\phi_{h}^{n+1}-\phi_{h}^{n-1}}{2\delta t},\Upsilon \right), \end{array}$$

(51) $$\begin{array}{cc} \; & U^{n+1}-U^{n-1}=\frac{1}{2}\left(H\left(\phi_{h}^{n}\right),\phi_{h}^{n+1}-\phi_{h}^{n-1}\right), \end{array}$$

with the initial conditions $\phi_{h}{|}_{t=0}=\phi_{h}^{0}$, $\psi_{h}{|}_{t=0}=0$, ${U|}_{t=0}=\sqrt{E\left(\phi_{h}^{0}\right)+B_{0}}$.

**Remark** **4.** *The MPFC model with long-range interaction is a sixth-order, nonlinear, damped wave equation, and the stiffness of the system exists in a higher-order linear operator. The stiff terms of the proposed scheme are treated implicitly, and the non-stiff terms are treated explicitly. Then, we can use the fast Fourier transform to directly invert the equation, so that we can obtain the numerical solution of the equation easily and quickly.*

**Remark** **5.** *In order to obtain the second-order accuracy of the fully discrete CNLF scheme, we need to compute all the initial values at $t=t^{1}$, which can be derived by applying the first-order backward Euler and corrector methods to obtain $\left(\phi_{h}^{1},\psi_{h}^{1},U^{1}\right)$. For ∀Θ,Λ and $\Upsilon \in S_{h}$, we obtain:*

(52) $$\begin{array}{cc} \; & \tilde{\beta }\left(\frac{{\tilde{\psi }}_{h}^{1}-\psi_{h}^{0}}{\delta t},\Theta \right)-\beta \left({\tilde{\psi }}_{h}^{1},\Theta \right)=M\left(\Delta \mu_{h}^{1}-\sigma \left(\phi_{h}^{1}-{\bar{\phi }}_{h}^{1}\right),\Theta \right), \end{array}$$

(53) $$\begin{array}{cc} \; & \left(\mu_{h}^{1},\Lambda \right)=\left({\left(1+\Delta \right)}^{2}\phi_{h}^{1}+H\left(\phi_{h}^{0}\right){\tilde{U}}^{1},\Lambda \right), \end{array}$$

(54) $$\begin{array}{cc} \; & \left({\tilde{\psi }}_{h}^{1},\Upsilon \right)=\left(\frac{\phi_{h}^{1}-\phi_{h}^{0}}{\delta t},\Upsilon \right) \end{array}$$

(55) $$\begin{array}{cc} \; & \frac{{\tilde{U}}^{1}-U^{0}}{\delta t}=\frac{1}{2}\int_{\Omega }H\left(\phi_{h}^{0}\right)\frac{\phi_{h}^{1}-\phi_{h}^{0}}{\delta t}dx. \end{array}$$

*Then, the corrector method is used as follows:*

(56) $$\begin{array}{cc} \; & \tilde{\beta }\left(\frac{\psi_{h}^{1}-\psi_{h}^{0}}{\delta t},\Theta \right)+\beta \left(\frac{\psi_{h}^{1}+\psi_{h}^{0}}{2},\Theta \right)=M\left(\Delta \mu_{h}^{\frac{1}{2}}-\sigma \left(\phi_{h}^{\frac{1}{2}}-{\bar{\phi }}_{h}^{\frac{1}{2}}\right),\Theta \right), \end{array}$$

(57) $$\begin{array}{cc} \; & \left(\mu_{h}^{\frac{1}{2}},\Lambda \right)=\left({\left(1+\Delta \right)}^{2}\frac{\phi_{h}^{1}+\phi_{h}^{0}}{2}+H\left({\tilde{\phi }}_{h}^{1}\right)\frac{U^{1}+U^{0}}{2},\Lambda \right) \end{array}$$

(58) $$\begin{array}{cc} \; & \left(\frac{\psi_{h}^{1}+\psi_{h}^{0}}{2},\Upsilon \right)=\left(\frac{\phi_{h}^{1}-\phi_{h}^{0}}{\delta t},\Upsilon \right) \end{array}$$

(59) $$\begin{array}{cc} \; & \frac{U^{1}-U^{0}}{\delta t}=\frac{1}{2}\int_{\Omega }H\left({\tilde{\phi }}_{h}^{1}\right)\frac{\phi_{h}^{1}-\phi_{h}^{0}}{\delta t}dx, \end{array}$$

*where $\phi_{h}^{\frac{1}{2}}=\frac{1}{2}\phi_{h}^{1}+\frac{1}{2}\phi_{h}^{0}$ and ${\bar{\phi }}_{h}^{\frac{1}{2}}=\frac{1}{\left|\Omega \right|}\int_{\Omega }\phi_{h}^{\frac{1}{2}}dx$. In addition, $\left(\phi_{h}^{0},\psi_{h}^{0},U^{0}\right)$ is given by the initial conditions for the MPFC model with long-range interaction.*

### 3.3. Mass Conservation and Unconditional Stability

In this subsection, we demonstrate the unconditional stability and mass conservation for the second-order fully discrete scheme (48)–(51).

We first show that the property of mass conservation still holds. Letting Θ=1 in (48) and (56), we obtain:

(60) $$\begin{array}{c} \int_{\Omega }\psi_{h}^{n+1}dx=\int_{\Omega }\psi_{h}^{n}dx=\int_{\Omega }\psi_{h}^{n-1}dx=\cdots =\int_{\Omega }\psi_{h}^{1}dx=\int_{\Omega }\psi_{h}^{0}dx=0. \end{array}$$

Letting Υ=1 in (50) and (58), we obtain:

(61) $$\begin{array}{c} \int_{\Omega }\phi_{h}^{n+1}dx=\int_{\Omega }\phi_{h}^{n}dx=\int_{\Omega }\phi_{h}^{n-1}dx=\cdots =\int_{\Omega }\phi_{h}^{1}dx=\int_{\Omega }\phi_{h}^{0}dx. \end{array}$$

Then, we establish the unconditional stability in the following theorem.

**Theorem** **2.** *The proposed scheme (48)–(51) is unconditionally stable in the sense that for any N≥2*

(62) $$\begin{array}{cc} \; & E_{CNLF}^{N-\frac{1}{2}}++\frac{\delta t\beta }{2M}\sum_{n=1}^{N-1}{∥\nabla \left(Q_{h}^{n+1}+Q_{h}^{n-1}\right)∥}^{2}=E_{CNLF}^{\frac{1}{2}}, \end{array}$$

*where the discrete energy $E_{CNLF}^{N-\frac{1}{2}}$*

(63) $$\begin{array}{cc} E_{CNLF}^{N-\frac{1}{2}}= & \frac{1}{2}\left(∥\left(1+\Delta \right)\phi_{h}^{N}{∥}^{2}+{∥\left(1+\Delta \right)\phi_{h}^{N-1}∥}^{2}\right)+\frac{\tilde{\beta }}{2M}\left(∥\nabla Q_{h}^{N}{∥}^{2}+{∥\nabla Q_{h}^{N-1}∥}^{2}\right)\\ \; & +{\left(U^{N}\right)}^{2}+{\left(U^{N-1}\right)}^{2}+\frac{\sigma }{2}\left(∥\phi_{h}^{N}-{\bar{\phi }}_{h}^{N}{∥}_{-1}^{2}+{∥\phi_{h}^{N-1}-{\bar{\phi }}_{h}^{N-1}∥}_{-1}^{2}\right). \end{array}$$

**Proof.**  Applying the $\Delta^{-1}$ operator to the formula (48) and taking $\Theta =\frac{\phi_{N}^{n+1}-\phi_{N}^{n-1}}{M}$ in (48) and $\Lambda =\phi_{N}^{n+1}-\phi_{N}^{n-1}$ in (49), we obtain:

(64) $$\begin{array}{cc} \; & \frac{\tilde{\beta }}{2M\delta t}\left(\Delta^{-1}\left(\psi_{h}^{n+1}-\psi_{h}^{n-1}\right),\phi_{h}^{n+1}-\phi_{h}^{n-1}\right)+\frac{\beta }{2M}\left(\Delta^{-1}\left(\psi_{h}^{n+1}+\psi_{h}^{n-1}\right),\phi_{h}^{n+1}-\phi_{h}^{n-1}\right)\\ \; & =\left(\mu_{h},\phi_{h}^{n+1}-\phi_{h}^{n-1}\right)-\sigma \left(\Delta^{-1}\left(\frac{\phi_{h}^{n+1}+\phi_{h}^{n-1}}{2}-\frac{{\bar{\phi }}_{h}^{n+1}+{\bar{\phi }}_{h}^{n-1}}{2}\right),\phi_{h}^{n+1}-\phi_{h}^{n-1}\right), \end{array}$$

(65) $$\begin{array}{c} \left(\mu_{h},\phi_{h}^{n+1}-\phi_{h}^{n-1}\right)=\left(\left(1+\Delta \right)\frac{\phi_{h}^{n+1}+\phi_{h}^{n-1}}{2},\left(1+\Delta \right)\left(\phi_{h}^{n+1}-\phi_{h}^{n-1}\right)\right)+\left(H\left(\phi_{h}^{n}\right)\frac{U^{n+1}+U^{n-1}}{2},\phi_{h}^{n+1}-\phi_{h}^{n-1}\right). \end{array}$$

By multiplying (51) with $\left(U^{n+1}+U^{n-1}\right)$, we have:

(66) $$\begin{array}{c} {\left(U^{n+1}\right)}^{2}-{\left(U^{n-1}\right)}^{2}=\frac{1}{2}H\left(\phi_{h}^{n}\right)\left(\phi_{h}^{n+1}-\phi_{h}^{n-1},U^{n+1}+U^{n-1}\right). \end{array}$$

We define $Q_{h}^{n+1}=\Delta^{-1}\psi_{h}^{n+1}$. By subtracting with the (n−1)th step, we can obtain:

(67) $$\begin{array}{c} \Delta \left(Q_{h}^{n+1}-Q_{h}^{n-1}\right)=\psi_{h}^{n+1}-\psi_{h}^{n-1}. \end{array}$$

Hence, using (67) and taking Υ=1 in (50), we derive:

(68) $$\begin{array}{cc} \; & \frac{\tilde{\beta }}{2\delta tM}\left(\Delta^{-1}\left(\psi_{h}^{n+1}-\psi_{h}^{n-1}\right),\phi_{h}^{n+1}-\phi_{h}^{n-1}\right)=-\frac{\tilde{\beta }}{2M}\left(∥\nabla Q_{h}^{n+1}{∥}^{2}-{∥\nabla Q_{h}^{n-1}∥}^{2}\right). \end{array}$$

(69) $$\begin{array}{cc} \; & \frac{\beta }{2M}\left(\Delta^{-1}\left(\psi_{h}^{n+1}+\psi_{h}^{n-1}\right),\phi_{h}^{n+1}-\phi_{h}^{n-1}\right)=-\frac{\delta t\beta }{2M}{∥\nabla \left(Q_{h}^{n+1}+Q_{h}^{n-1}\right)∥}^{2}. \end{array}$$

Since $\int_{\Omega }\left(\phi_{h}^{n+1}-{\bar{\phi }}_{h}^{n+1}\right)dx=0$, i.e., $\phi_{h}^{n+1}-{\bar{\phi }}_{h}^{n+1}\in L_{0}^{2}\left(\Omega \right)$, by setting $\kappa_{h}^{n}={\left(-\Delta \right)}^{-1}\left(\phi_{h}^{n}-{\bar{\phi }}_{h}^{n}\right)$, $\kappa_{h}^{n}\in H_{per}^{2}\left(\Omega \right)\cap L_{0}^{2}\left(\Omega \right)$, and notice that $(\kappa_{h}^{n},1)=0$. We obtain:

(70) $$\begin{array}{cc} \; & \sigma \left({\left(-\Delta \right)}^{-1}(\frac{\phi_{h}^{n+1}+\phi_{h}^{n-1}}{2}-\frac{{\bar{\phi }}_{h}^{n+1}+{\bar{\phi }}_{h}^{n-1}}{2}),\phi_{h}^{n+1}-\phi_{h}^{n-1}\right)\\ \; & =\frac{\sigma }{2}\left(\kappa_{h}^{n+1}+\kappa_{h}^{n-1},-\Delta \left(\kappa_{h}^{n+1}-\kappa_{h}^{n-1}\right)\right)dx\\ \; & =\frac{\sigma }{2}∥\phi_{h}^{n+1}-{\bar{\phi }}_{h}^{n+1}{∥}_{-1}^{2}-\frac{\sigma }{2}{∥\phi_{h}^{n-1}-{\bar{\phi }}_{h}^{n-1}∥}_{-1}^{2}. \end{array}$$

Therefore, by combining (64)–(70), we arrive at:

(71) $$\begin{array}{cc} \; & \frac{1}{2}\left(∥\left(1+\Delta \right)\phi_{h}^{n+1}{∥}^{2}+{∥\left(1+\Delta \right)\phi_{h}^{n}∥}^{2}\right)-\left(∥\left(1+\Delta \right)\phi_{h}^{n}{∥}^{2}+{∥\left(1+\Delta \right)\phi_{h}^{n-1}∥}^{2}\right)+\frac{\tilde{\beta }}{2M}\left(∥\nabla Q_{h}^{n+1}{∥}^{2}+{∥\nabla Q_{h}^{n}∥}^{2}\right)\\ \; & -\frac{\tilde{\beta }}{2M}\left(∥\nabla Q_{h}^{n}{∥}^{2}+{∥\nabla Q_{h}^{n-1}∥}^{2}\right)+\frac{\sigma }{2}\left(∥\phi_{h}^{n+1}-{\bar{\phi }}_{h}^{n+1}{∥}_{-1}^{2}+{∥\phi_{h}^{n}-{\bar{\phi }}_{h}^{n}∥}_{-1}^{2}\right)+\left({\left(U^{n+1}\right)}^{2}+{\left(U^{n}\right)}^{2}\right)\\ \; & -\left({\left(U^{n}\right)}^{2}+{\left(U^{n-1}\right)}^{2}\right)-\frac{\sigma }{2}\left(∥\phi_{h}^{n}-{\bar{\phi }}_{h}^{n}{∥}_{-1}^{2}+{∥\phi_{h}^{n-1}-{\bar{\phi }}_{h}^{n-1}∥}_{-1}^{2}\right)+\frac{\delta t\beta }{2M}{∥\nabla \left(Q_{h}^{n+1}+Q_{h}^{n-1}\right)∥}^{2}=0. \end{array}$$

Then, summing up (71) from n=1 to n=N−1, we have:

(72) $$\begin{array}{cc} \; & \frac{1}{2}\left(∥\left(1+\Delta \right)\phi_{h}^{N}{∥}^{2}+{∥\left(1+\Delta \right)\phi_{h}^{N-1}∥}^{2}\right)+\frac{\tilde{\beta }}{2M}\left(∥\nabla Q_{h}^{N}{∥}^{2}+{∥\nabla Q_{h}^{N-1}∥}^{2}\right)+{\left(U^{N}\right)}^{2}+{\left(U^{N-1}\right)}^{2}\\ \; & +\frac{\sigma }{2}\left(∥\phi_{h}^{N}-{\bar{\phi }}_{h}^{N}{∥}_{-1}^{2}+{∥\phi_{h}^{N-1}-{\bar{\phi }}_{h}^{N-1}∥}_{-1}^{2}\right)+\frac{\delta t\beta }{2M}\sum_{n=1}^{n=N-1}{∥\nabla \left(Q_{h}^{n+1}+Q_{h}^{n-1}\right)∥}^{2}\\ = & -\left(∥\left(1+\Delta \right)\phi_{h}^{1}{∥}^{2}+{∥\left(1+\Delta \right)\phi_{h}^{0}∥}^{2}\right)+\frac{\tilde{\beta }}{2M}\left(∥\nabla Q_{h}^{1}{∥}^{2}+{∥\nabla Q_{h}^{0}∥}^{2}\right)+{\left(U^{1}\right)}^{2}+{\left(U^{0}\right)}^{2}\\ \; & +\frac{\sigma }{2}\left(∥\phi_{h}^{1}-{\bar{\phi }}_{h}^{1}{∥}_{-1}^{2}+{∥\phi_{h}^{0}-{\bar{\phi }}_{h}^{0}∥}_{-1}^{2}\right). \end{array}$$

Therefore, we can complete the proof for the desired result (62). □

As a direct consequence of the unconditional stability, a uniform-in-time $H^{2}$ bound for the numerical solution is derived as follows.

**Lemma** **3.** *Let $\phi_{h}^{n}$ be the solution of (48)–(51). $C_{0}$ and $C_{1}$ are independent of δt and h. Suppose that the initial data are sufficiently regular such that*

(73) $$\begin{array}{cc} \; & \left(∥\left(1+\Delta \right)\phi_{h}^{1}{∥}^{2}+{∥\left(1+\Delta \right)\phi_{h}^{0}∥}^{2}\right)+\frac{\tilde{\beta }}{2M}\left(∥\nabla Q_{h}^{1}{∥}^{2}+{∥\nabla Q_{h}^{0}∥}^{2}\right)\\ \; & +{\left(U^{1}\right)}^{2}+{\left(U^{0}\right)}^{2}+\frac{\sigma }{2}\left(∥\phi_{h}^{1}-{\bar{\phi }}_{h}^{1}{∥}_{-1}^{2}+{∥\phi_{h}^{0}-{\bar{\phi }}_{h}^{0}∥}_{-1}^{2}\right)\leq C_{0}, \end{array}$$

*Then, we have $∥\phi_{h}^{n}{∥}_{H^{2}}\leq C_{1}$ for all n and h.*

**Proof.**  As a result of (62), we have:

(74) $$\begin{array}{cc} ∥\left(1+\Delta \right)\phi_{h}^{n}{∥}^{2}\leq & \left(∥\left(1+\Delta \right)\phi_{h}^{1}{∥}^{2}+{∥\left(1+\Delta \right)\phi_{h}^{0}∥}^{2}\right)+\frac{\tilde{\beta }}{2M}\left(∥\nabla Q_{h}^{1}{∥}^{2}+{∥\nabla Q_{h}^{0}∥}^{2}\right)\\ \; & +{\left(U^{1}\right)}^{2}+{\left(U^{0}\right)}^{2}+\frac{\sigma }{2}\left(∥\phi_{h}^{1}-{\bar{\phi }}_{h}^{1}{∥}_{-1}^{2}+{∥\phi_{h}^{0}-{\bar{\phi }}_{h}^{0}∥}_{-1}^{2}\right)\leq C_{0}, \end{array}$$

for any n≥1. In addition,

(75) $$\begin{array}{c} ∥\left(1+\Delta \right)\phi_{h}^{n}{∥}^{2}=∥\phi_{h}^{n}+\Delta \phi_{h}^{n}{∥}^{2}\leq ∥\phi_{h}^{n}{∥}^{2}+{∥\Delta \phi_{h}^{n}∥}^{2}\leq C_{0}. \end{array}$$

Using integration by parts and Cauchy inequality yields:

(76) $$\begin{array}{c} ∥\nabla \phi_{h}^{n}{∥}^{2}=-\left(\phi_{h}^{n},\Delta \phi_{h}^{n}\right)\leq ∥\phi_{h}^{n}∥\cdot ∥\Delta \phi_{h}^{n}∥\leq \frac{1}{2}\left(∥\phi_{h}^{n}{∥}^{2}+{∥\Delta \phi_{h}^{n}∥}^{2}\right)\leq \frac{1}{2}C_{0}. \end{array}$$

Therefore, we obtain:

(77) $$\begin{array}{c} ∥\phi_{h}^{n}{∥}_{H^{2}}={\left(∥\phi_{h}^{n}{∥}^{2}+∥\nabla \phi_{h}^{n}{∥}^{2}+{∥\Delta \phi_{h}^{n}∥}^{2}\right)}^{\frac{1}{2}}\leq {\left(C_{0}+\frac{1}{2}C_{0}\right)}^{\frac{1}{2}}\leq C_{1}. \end{array}$$

This completes the proof of Lemma 3. □

## 4. Numerical Experiments

In this section, we perform various numerical simulations to test the accuracy, stability, and efficiency of the CNLF scheme (48)–(51) for solving the MPFC problem with long-range interaction and show the energy stability. We mainly use the SAV approach in time and the Fourier spectral method in space; in addition, the fast Fourier transform is applied for all numerical simulations.

### 4.1. Convergence Test

We firstly verify the temporal convergence rates of the presented scheme (48)–(51). We allocate 128 Fourier modes in each direction, and the numerical simulation domain is set as ${\left[0,\;128\right]}^{2}$. Some parameters are commonly taken to be $M=1,\;\tilde{\beta }=100,\;\sigma =100,$ β=0.03, $T=1,\;\epsilon =0.025,\;B_{0}=\epsilon^{2}\left|\Omega \right|$. In addition, we select the appropriate forcing term such that the following manufactured solution is provided by

(78) $$\begin{array}{cc} \; & \phi \left(x,y,t\right)=sin\left(\frac{2\pi }{64}x\right)cos\left(\frac{2\pi }{64}y\right)cos\left(t\right). \end{array}$$

**Figure 1:**
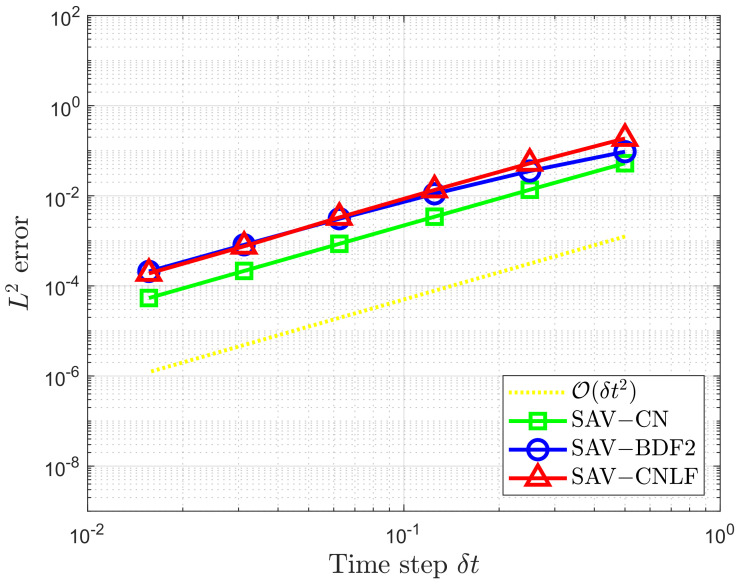
The $L^{2}$ errors of the phase variable ϕ for the SAV-CN, SAV-BDF2, and SAV-CNLF schemes.

**Figure 2:**
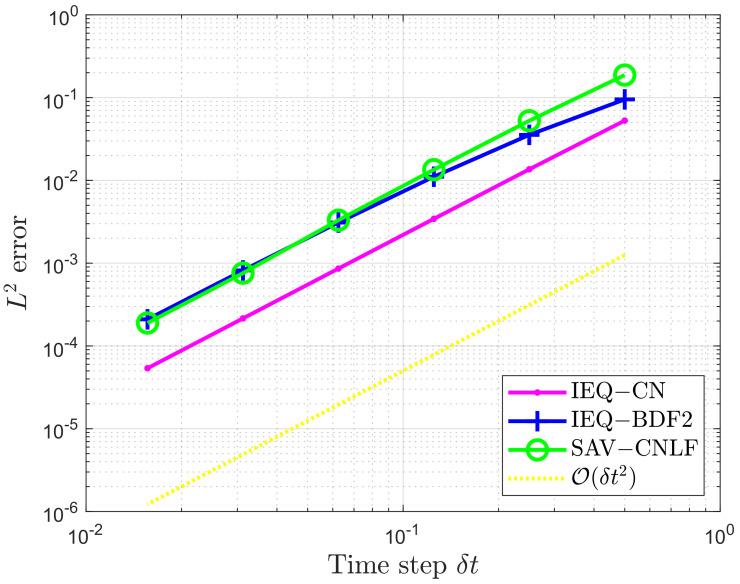
The $L^{2}$ errors of the phase variable ϕ for the IEQ-CN, IEQ-BDF2, and SAV-CNLF schemes.

In order to verify the accuracy and efficiency of the proposed CNLF scheme, we show the $L^{2}$ errors at T=1 of the phase variable ϕ between the numerical solution and the exact solution in Figure 1 and Figure 2. Based on the same parameters, we compare the temporal convergence rates of the Crank–Nicolson scheme described by SAV-CN. The BDF2 scheme is described by SAV-BDF2 [17], and the second-order Crank–Nicolson and BDF2 schemes proposed based on the IEQ approach are denoted by IEQ-CN and IEQ-BDF2 [33], respectively.

In Figure 1, it is easy to observe that the SAV-CNLF, SAV-CN, and SAV-BDF2 schemes are stable for all tested time steps and result in good approximations and corresponding orders of accuracy. This further proves that our scheme is second-order stable.

When using the IEQ method, we need to solve the linear system with variable coefficients at each time step. On the contrary, when using the SAV method, we only need to solve two linear systems with constant coefficients at each time step. Therefore, although IEQ-CN, IEQ-BDF2, and SAV-CNLF all have good approximation effects in Figure 2, the total CPU time including all various time step δt of the IEQ-CN and IEQ-BDF2 schemes is 2.88 s and 3.25 s, respectively, and the SAV-CNLF scheme only needs 0.94 s. That is, the IEQ-CN and IEQ-BDF2 schemes require more CPU time than SAV-CNLF. Hence, we can find that the SAV approach proposed in this paper is more effective than the IEQ approach. Combining the two figures, we can verify that our proposed scheme is accurate, effective, and easy to implement.

### 4.2. Energy Test

In order to verify the energy stability of the CNLF scheme (48)–(51), the smooth initial data ϕ are given by:

(79) $$\begin{array}{cc} \phi^{0}\left(x,y\right) & =0.07-0.02cos\left(\frac{2\pi (x-12)}{32}\right)sin\left(\frac{2\pi (y-1)}{32}\right)\\ \; & +0.02cos^{2}\left(\frac{\pi (x+10)}{32}\right)cos^{2}\left(\frac{\pi (y+3)}{32}\right)-0.01sin^{2}\left(\frac{4x\pi }{32}\right)sin^{2}\left(\frac{4\pi (y-6)}{32}\right). \end{array}$$

**Figure 3:**
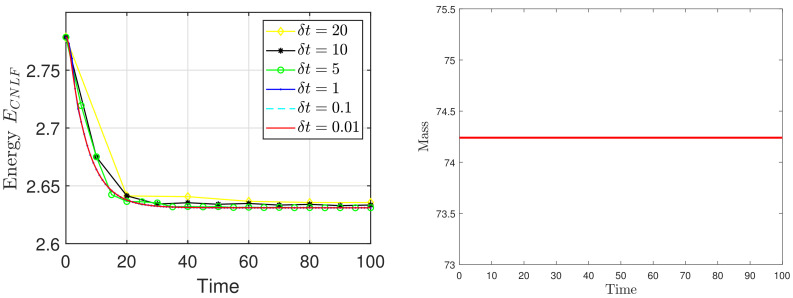
(**left**) The curves of the energy $E_{CNLF}$ with different time step sizes. (**right**) The curves of the mass.

**Figure 4:**
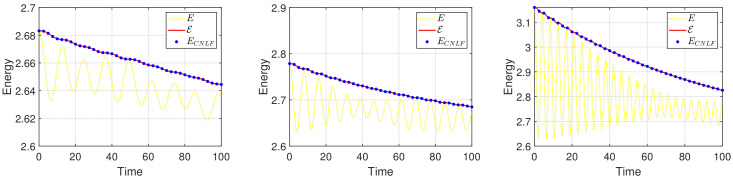
The curves of energy *E*, pseudo energy ε, and modified discrete energy $E_{CNLF}$ with different parameters σ=0, σ=0.1, σ=0.5 in sequence.

**Figure 5:**
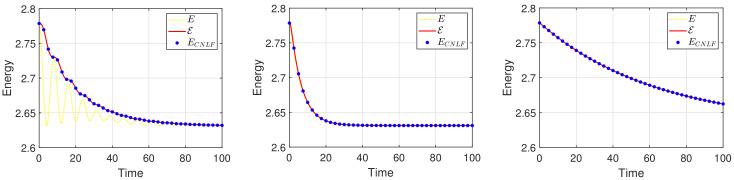
The curves of energy *E*, pseudo energy ε, and modified discrete energy $E_{CNLF}$ with different parameters β=0.05, β=2, β=20 in sequence.

**Figure 6:**
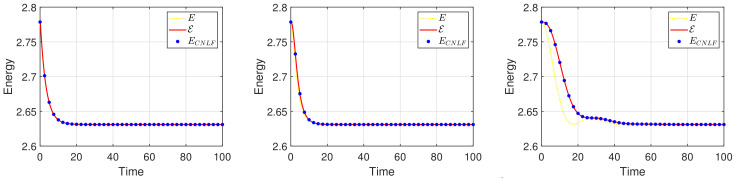
The curves of energy *E*, pseudo energy ε, and modified discrete energy $E_{CNLF}$ with different parameters $\tilde{\beta }=0.1$, $\tilde{\beta }=1$, $\tilde{\beta }=10$ in sequence.

We set the appropriate parameters, which are ϵ=0.025, M=1, T=100, and the computational domain is $\Omega ={\left[0,\;32\right]}^{2}$. For spatial discretization, the $128^{2}$ Fourier modes are used. In Figure 3, we display the evolutionary process of the energy $E_{CNLF}$ with different time steps δt=0.01, 0.1, 1, 5, 10, 20, where the other parameters are $\tilde{\beta }=1$, β=2, σ=0.05. It means that the energy $E_{CNLF}$ is still decreasing over time at large steps. Meanwhile, we show that the mass is invariant over time. In Figure 4, we show how the evolutionary process of original energy *E*, pseudo energy ε, and modified discrete energy $E_{CNLF}$ changes over time with σ=0, σ=0.1, and σ=0.5, respectively, where β=0.01, $\tilde{\beta }=1$, δt=0.01. We discover that ε and $E_{CNLF}$ are decreasing in time, while the original energy *E* is becoming more perturbed as σ becomes bigger. That indicates the strength of the long-range interaction between the chain molecules is stronger and the vibrations are more intense. In addition, we show how the evolution of original energy *E*, pseudo energy ε, and modified discrete energy $E_{CNLF}$ changes over time with β=0.05, β=2, and β=20, respectively, where σ=0.1, δt=0.01 in Figure 5. We observe that the behaviors of the MPFC model are consistent with the PFC model and ε is the same as *E* when β is large (β = 20, in the high damping case). In particular, when β is small (β = 0.05, lower damping case), *E* shows an oscillatory behavior, and ε differs from *E*. In Figure 6, we depict the changes in the three energies with different $\tilde{\beta }$ using the same parameters σ=0.1, β=1. It can be observed that the energy has a great performance. Due to the second-order time derivative of ϕ giving rise to short wavelength oscillations, the original energy *E* has a little oscillation as $\tilde{\beta }$ increases. As shown in the above figures, the discrete energy $E_{CNLF}$ of the CNLF scheme is non-increasing in time.

### 4.3. Phase Transition Behaviors in 2D and 3D

In this subsection, we display the phase transition behaviors of the density field by using the second-order CNLF scheme in 2D and 3D, respectively.

**Figure 7:**
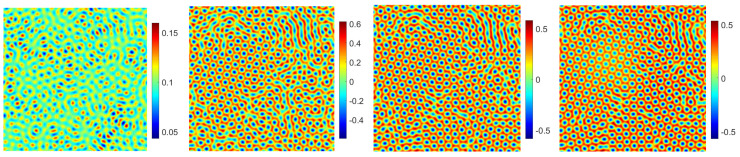
The evolutionary process of the phase transition behaviors of the phase variable ϕ with t=20,80,150,200 in sequence with σ=0.1, $\tilde{\beta }=0.1$.

**Figure 8:**
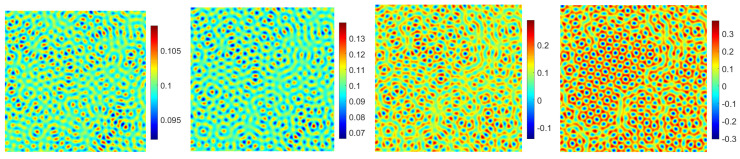
The evolutionary process of the phase transition behaviors of the phase variable ϕ with t=20,80,150,200 in sequence with σ=0.2, $\tilde{\beta }=0.2$.

**Figure 9:**
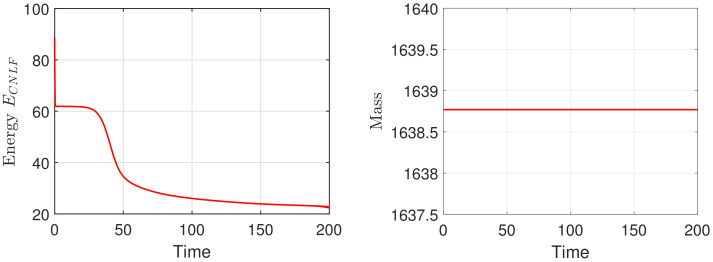
The evolutionary process of the energy $E_{CNLF}$ (**left**) and corresponding mass (**right**) for phase variable with σ=0.1, $\tilde{\beta }=0.1$ and the time step δt=0.0005.

**Figure 10:**
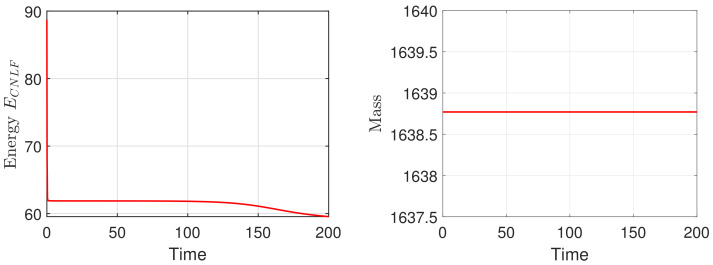
The evolutionary process of the energy $E_{CNLF}$ (**left**) and corresponding mass (**right**) for phase variable with σ=0.2, $\tilde{\beta }=0.2$ and the time step δt=0.0005.

Firstly, we mainly simulate the phase transition behaviors with time evolution. In the 2D case, with the numerical simulation domain of ${\left[0,\;128\right]}^{2}$, the mesh size is h=1. For the proposed scheme (48)–(51), we select the initial data $\phi^{0}=0.1+rand\left(\right)$, where rand() is a uniformly distributed random number between −0.01 and 0.01 at the grid points in 2D. In addition, the fixed parameters are set as ϵ=0.25,T=200,δt=0.0005,M=1,β=1. In Figure 7 and Figure 8, we display the evolutionary process of the phase transition with σ=0.1,0.2 and $\tilde{\beta }=0.1,0.2$ at t=20,80,150,200. Moreover, one can see that under the appropriate parameters, the pattern will become hexagonal over time. The numerical results are consistent with the transition behavior in [26,34]. With the decrease in σ and $\tilde{\beta }$, the phase variable reaches the steady-state faster and makes the steady-state pattern of the phase transition more uniform. Finally, we show the corresponding energy and mass associated with the phase variable ϕ in Figure 9 and Figure 10, respectively. We demonstrate that the energy-stable and mass-conserving properties of the proposed scheme are consistent with the theoretical result.

**Figure 11:**
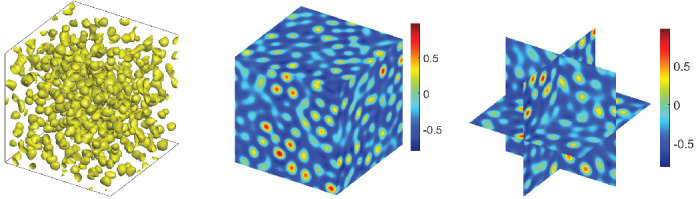
The evolutionary process of the phase transition behaviors of the phase variable ϕ in 3D with $\tilde{\beta }=0.1$.

**Figure 12:**
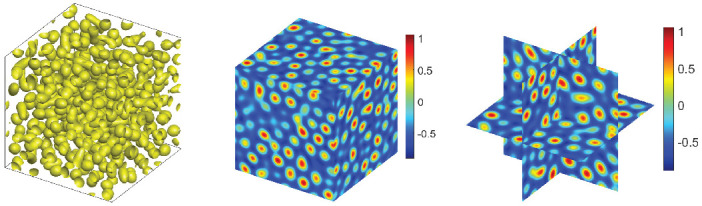
The evolutionary process of the phase transition behaviors of the phase variable ϕ in 3D with $\tilde{\beta }=0.2$.

**Figure 13:**
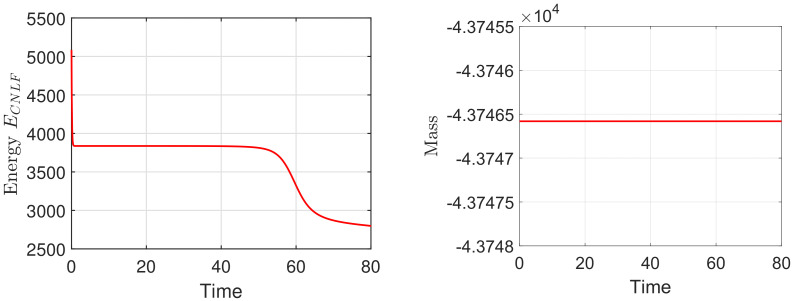
The evolutionary process of the energy $E_{CNLF}$ (**left**) and corresponding mass (**right**) for phase variable with the time step δt=0.0005.

For the 3D simulation, we firstly set the numerical simulation domain ${\left[0,\;50\right]}^{3}$ and use $64^{3}$ Fourier modes. For the CNLF scheme, we set the appropriate initial data $\phi^{0}=-0.35+rand\left(\right)$, where rand() is a uniformly distributed random number between −0.01 and 0.01 at the grid points in 3D. In addition, the parameters are chosen as ϵ=0.56,T=80,δt=0.0005,M=1,σ=0.1,β=1. In Figure 11 and Figure 12, we show the steady-state microstructure of the phase transition behaviors with $\tilde{\beta }=0.1,0.2$, and we can obtain similar results as the energy $E_{CNLF}$ and mass in Figure 13. It can be seen that the pattern becomes a more uniform lattice structure from stripes and the steady-state microstructure of the phase transition behaviors is achieved faster as the value of $\tilde{\beta }$ increases. All of the numerical results are consistent with the transition behavior in [26,34]. In addition, the curves depict that the free energy is monotonically non-increasing over time.

## 5. Conclusions

In this paper, we have presented an efficient, fully discrete and unconditionally stable CNLF scheme for the MPFC equation with long-range interaction. Our proposed scheme conquer the inconvenience from nonlinearities by linearizing the nonlinear cubic term, and the advantage of the presented scheme is that, in each time step, only two decoupled six-order linear equations with constant coefficients need to be solved. Thus, it is easy to implement and efficient. In addition, we established the unique solvability at the semi-discrete level, and we rigorously proved the mass conservation and unconditional stability at the fully discrete level. Finally, numerical results for some benchmark simulations were presented to verify the efficiency and stability of the developed scheme.

Overall, the proposed CNLF scheme is accurate, efficient, and easy to implement, and the presented idea can be readily extended to study a broader class of multiphase hydrodynamic models for developing fully discrete, linear, and unconditionally stable schemes.
